# Identifying pain trajectories in children and youth with cerebral palsy: a pilot study

**DOI:** 10.1186/s12887-021-02861-3

**Published:** 2021-09-29

**Authors:** Heather M. Shearer, Pierre Côté, Sheilah Hogg-Johnson, Patricia McKeever, Darcy L. Fehlings

**Affiliations:** 1 Institute of Health Policy, Management and Evaluation, University of Toronto, Toronto, Canada; 2 Bloorview Research Institute, Holland Bloorview Kids Rehabilitation Hospital, 150 Kilgour Rd., Toronto, ON M4G 1R8 Canada; 3 Institute for Disability and Rehabilitation Research, Oshawa, Ontario Canada; 4 Faculty of Health Sciences, Ontario Tech University, Oshawa, Canada; 5 Dalla Lana School of Public Health, University of Toronto, Toronto, Canada; 6 Research and Innovation, Canadian Memorial Chiropractic College, Toronto, Canada; 7 Bloomberg Faculty of Nursing, University of Toronto, Toronto, Canada; 8 Department of Paediatrics, University of Toronto, Toronto, Canada

**Keywords:** Cerebral palsy, Pain, Pilot study, Child, Youth, Adolescent

## Abstract

**Background:**

Although chronic pain is common in children with cerebral palsy (CP), little is known about short-term pain fluctuations and their impact on children’s well-being. High-quality cohort studies are needed to understand the clinical course of pain in this population. We aimed to determine the feasibility of conducting a multicentre cohort study. In this pilot study we assessed: 1) study processes, 2) resource and 3) management indicators including recruitment and follow-up rates, data completeness, participant characteristics, and successes and barriers in the study conduct.

**Methods:**

A multi-centre pilot cohort study was conducted with 10 Canadian children/youth with CP attending one of two children’s rehabilitation centers. We collected self-reported pain intensity (Faces Pain Scale-Revised [FPS-R], Numeric Rating Scale [NRS]); pain interference (PROMIS PI); pain location (pain diagram); physical and psychological well-being (KIDSCREEN-27), sleep characteristics, preceding months’ interventions, and some clinical characteristics at baseline. Average pain intensity was reported weekly for five weeks. Well-being, sleep and interventions were measured at baseline and again at five weeks. We used feasibility indicators to evaluate:1) study processes (e.g. recruitment, attrition rates); 2) resources (e.g. data completion, budgetary challenges); and 3) management (e.g. data optimization, variability of participants and pain scores).

**Results:**

Between March and May 2019, 24 children and their parents/guardians were contacted and 20 met eligibility criteria. Of those, 10 agreed to in-person screening (50%) and were subsequently enrolled. The follow-up rate was 90% and self-reported missing data was minimal. Ninety percent of participants chose e-questionnaire follow-ups versus mailed paper questionnaires. Sixty percent required reminders to complete e-follow-ups. Participants were aged 8-17 years, five were female, GMFCS levels I-IV (none with level V), 90% had spastic CP and 80% reported having pain in the preceding week. Pain intensity (FPS-R) between participants ranged from 0-8/10 at baseline and 0-6/10 across all four weekly follow-ups.

**Conclusions:**

This pilot study demonstrates the feasibility of conducting a multicentre cohort study to identify short-term pain trajectories and measure their association with well-being in children and youth with CP. Additional strategies to improve recruitment and accessibility for those with GMFCS levels V should be implemented in future studies.

## Background

Cerebral palsy (CP) is the most common motor disability in children and youth, with an incidence of approximately 2 per 1000 live births [1, 2]. It is associated with complex, life-long consequential health challenges and comorbidities.

Pain is experienced by 14% to 76% of children and youth with CP [3–6] and 25% have moderate to severe chronic pain restricting daily activities [7]. This is significant because pain is the main risk factor for poor well-being [8–10]. Evidence from one five-year cohort study suggests that pain increases from childhood to adolescence [8]. Pain is also associated with diminished physical, psychological, and social well-being and overall quality of life [8]. Other studies suggest that pain is associated with poorer psychological well-being, psychological disorders and depressive symptoms [9, 11]. One cross-sectional study suggests that children with severe pain were over 2.5 times more likely to report psychological symptoms than those without severe pain [12].

Measuring self-reported pain intensity can be challenging. Clinicians and researchers must consider the validity and reliability of scales and their applicability based on individuals’ type of pain and cognitive development. In a recent systematic review aimed to identify the measurement properties of single-item self-report pain intensity scales, Birnie et al. (2019) reported that only the Numeric Rating Scale (NRS), Faces Pain Scale-Revised (FPS-R), and the Color Analogue Scale (CAS) were strongly recommended for self-reporting acute pain by children aged 8-18 [13]. Furthermore, based on the available evidence, no measures met the criteria for strong recommendation to assess chronic pain in children or adolescents [13]. Depending on a child’s medical and developmental complexity, these scales may not be appropriate. Consideration of those unable to verbally or physically report pain and its characteristics is important. Alternate approaches to pain measurement may be required and methodology should be appropriately tailored to the clinical or research environment. Understanding pain etiology is important, especially among children who may be unable to express themselves or have cognitive delays. Some common causes of pain in children/youth with CP, that were physician-identified, include hip dislocation/subluxation, sub-type of CP (e.g. dystonia), musculoskeletal deformity and gastro-intestinal issues (e.g. constipation, gastroesophageal reflux) [6].

To date, little is known about short-term pain fluctuations and their effects on the well-being in these children. It is necessary to fill this knowledge gap to prevent pain chronicity, identify effective treatments and mitigate its impact on well-being. However, conducting cohort studies in this population requires careful planning and the feasibility needs to be established to ensure that recruitment, enrollment, data collection and follow-up procedures are adequate to maximize the internal validity of a study. We conducted a pilot multi-center cohort study to evaluate the feasibility of a larger cohort study by measuring study processes, resources and management [14].

## Methods

### Feasibility indicators

Our pilot study assessed three feasibility indicators. First, *processes* assessed the feasibility of key methodological steps: 1) recruitment and participation; 2) attrition, and 3) implementation of inclusion criteria [14]. Second, *resources* assessed time and resource issues by measuring data completion and use of the research electronic data capture software (REDCap) [14]. Finally, we identified potential human and data issues by assessing data variability and measuring adverse events related to negative mood [14]. We were especially interested in identifying variability in pain intensity scores between participants and across time-points. Participant feedback of barriers to study success were reported within each category.

### Study design & settings

In February 2018, the lead author (HS) met with members of the Youth Advisory Council (age ≥14 years) at Holland Bloorview Kids Rehabilitation Hospital (Holland Bloorview) to discuss the proposed study design (participants, recruitment, questionnaires, as well as suitable transportation and honorariums). This feedback informed the conduct of the pilot study processes.

A multi-centre pilot cohort study was conducted at two children’s treatment centers (CTC) in Ontario, Canada: 1) Holland Bloorview, serving the greater Toronto area; and 2) Grandview Children's Centre (Grandview), serving the Durham region between March and June, 2019. In Ontario, young children diagnosed with CP under the age of 19 are referred to one of 21 designated CTCs.

### Study sample

The cohort included 10 children or youth, five from each site. To be included, eligible participants met the inclusion/exclusion criteria outlined in Table 1. With the aim of recruiting a wide variety of participants, children who communicated either verbally or non-verbally (with the use of assistive devices) were eligible for study recruitment. Additionally, language was not an exclusion criteria as an interpreter was available as needed.

**Table 1:** Inclusion and exclusion criteria for pilot study participants

**Inclusion Criteria**	
1. Clinical diagnosis of any form of CP 2. Age 8 to < 19 years old 3. Any GMFCS level 4. Successful completion of a sorting task^a^ 5. Willingness to discuss their pain/physical discomfort	
**Exclusion Criteria**	
1. Inability to communicate independently with or without assistive devices, irrespective of motor ability 2. Inability to complete electronic questionnaires, with or without assistive devices.	

GMFCS: Gross Motor Function Classification System. ^a^The sorting task was a multi-step procedure whereby children/youth had to: 1) sort 5 randomly lettered cubes from smallest to largest size; 2) match each cube to a picture scale (on paper); 3) state the name of something they enjoyed a lot, were neutral about, and disliked and then ranked how much they liked each on a 5-point Likert scale ranging from ‘not at all’ to ‘extremely’. Individuals had to complete all activities correctly to successfully pass the sorting task

### Recruitment

Study recruitment occurred between March 15 and May 8, 2019. We used multiple recruitment methods, including posters and electronic signage in both CTCs and circulating the poster through social media (approved Twitter and Facebook groups). At Holland Bloorview, we used an institutional and ethics-approved voluntary research participation call list. Physiotherapists from both sites distributed flyers and introduced the study to clients/parents with CP within the eligible age range. The lead author used a two-step screening process. She contacted clients/parents by phone to introduce the study, complete an initial telephone screen (identifying clients 8 to <19 years old, diagnosed with CP, and able to communicate) and book clients for in-person meetings. Next, potential participants met with the lead author to complete a sorting task and answer questions about communication data collection procedures. The sorting task was conducted to help determine participants’ developmental ability to complete the questionnaires, however it did not assess numeracy skills. If eligible, informed consent and assent was obtained and the client was enrolled.

### Study questionnaire

**Fig. 1:**
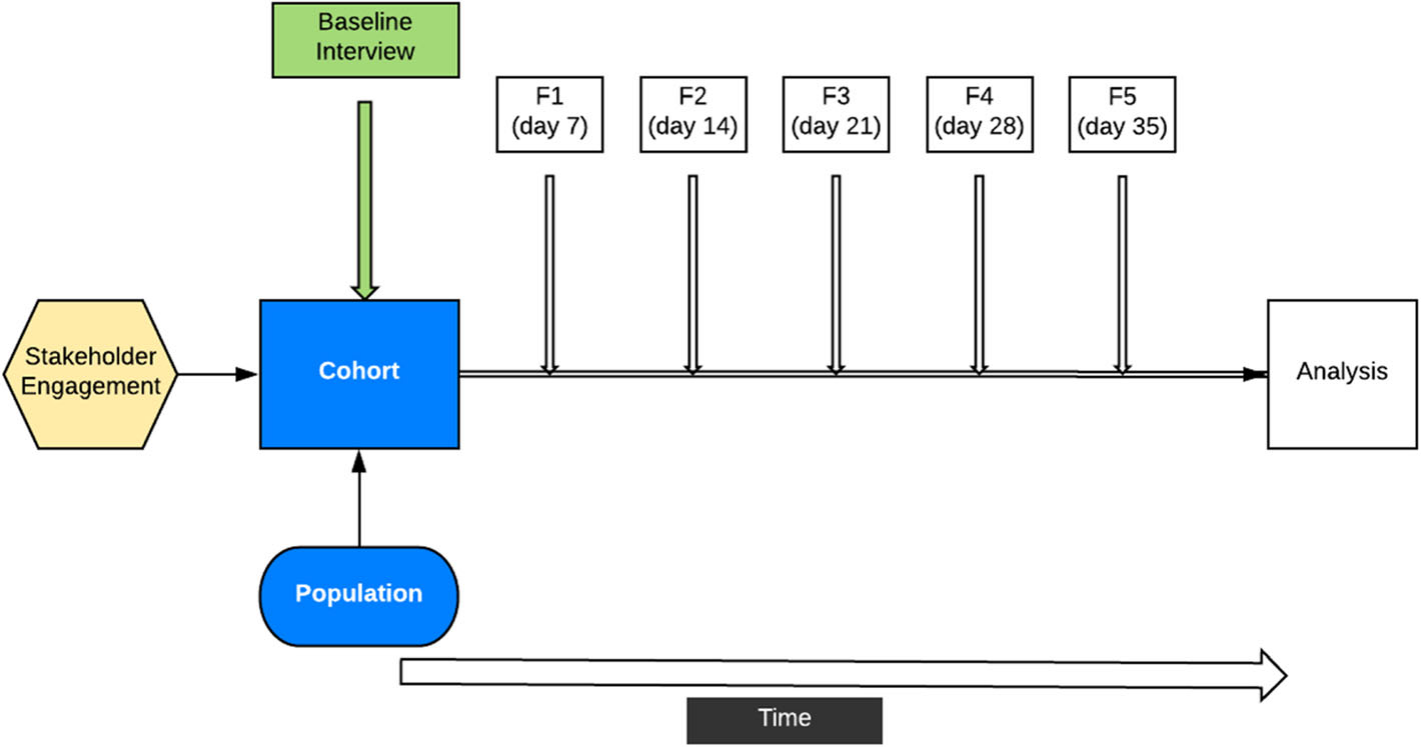
Pilot study schematic. F: follow-up. The schematic provides a visual representation of the study processes. First, we met with youth stakeholders to gain insight regarding study processes. Next, we recruited eligible participants from the CP populations attending two Ontario Children’s Treatment Centers and conducted screening and the baseline questionnaire. A short follow-up questionnaire was distributed electronically once/week for five weeks

We created baseline and follow-up electronic questionnaires using REDCap electronic data capture tools [15, 16] (Fig. 1).

The questionnaires included standardized self-report instruments. The baseline questionnaire asked participants to report their weekly pain including overall pain intensity, pain location and interference, and well-being. All responses to questionnaires were provided by the participating children/youth. Parents/guardians were instructed during the baseline meeting that throughout the study, responses must be provided by the child/youth, however, the parent/guardian could be asked or remind the child of pain episodes in the past week.

#### Pain intensity

Self-reported pain intensity was measured using the Faces Pain Scale-Revised (FPS-R) [17]. It is completed by children aged 4-18 years without a proxy and has good construct validity and responsiveness [17, 18]. It consists of six gender-neutral faces that depict ‘no pain’ to ‘most pain possible’ expressions, ordered numerically 0 to 10 [19]. The Numeric Rating Scale (NRS) (0=no pain, 10=worst pain ever), a preferred method for reporting pain intensity among some individuals, was used to assess potential misclassification of pain ratings on the FPS-R [20, 21].

#### Pain interference

The 8-item PROMIS Pediatric Short Form v2.0 pain interference questionnaire assessed the impact of pain on daily activities, physical functioning and socioemotional problems in the past week using a 5-point Likert scale (ranging from ‘never’ to ‘almost always’) [22, 23]. Higher scores indicate greater impairment (T-scores ranging from 34-78) [22, 24].

#### Physical and psychological well-being

The KIDSCREEN-27 measured well-being in the previous week. Twenty-seven items are scored on a 5-point Likert scale (ranging from 1=“not at all” to 5=“very much”), with higher scores indicating better quality of life. This scale is applicable to healthy and chronically ill children aged 8-18 years [25], has been used with CP populations [8, 26], and has good psychometric properties [25].

#### Demographic and clinical variables

These variables were collected from health records: age, sex, and socioeconomic status [based on postal code]. The following variables were also collected because they may be potential confounders in the larger cohort study: self-reported interventions in the month prior, sleep interference/disturbance using the PROMIS pediatric scales [27], CP diagnosis, motor function (GMFCS level), hip status, and presence of other health comorbidities.

#### Adverse events (AEs)

Measured by participant self-report and by monitoring KIDSCREEN-27 psychological well-being responses at baseline and five week follow-up. If participants reported very low mood scores (7/35), the lead author would contact the site-specific physician to speak with the participant and family members.

Our methodology did not include any formal qualitative data collection regarding completing the questionnaires or participating in the study.

### Data collection

After enrollment, participants completed the baseline questionnaire using a study laptop. Participants had the option to complete follow-up questionnaires through an emailed electronic link sent to themselves or their parent/caregiver or by completing paper questionnaires in pre-addressed, stamped return envelopes.

#### Follow-up

Participants were followed weekly for five weeks. Up to three automated electronic reminders were sent after 24 hours if follow-ups were not completed. HS telephoned participants if electronic reminders were not successful. Table 2 summarizes the data collection process.

**Table 2:** Measures used at baseline and follow-up questionnaires

Measures	Baseline	Day 7	Day 14	Day 21	Day 28	Day 35
Faces pain scale-revised	x	x	x	x	x	
Numeric Rating Scale	x	x	x	x	x	
Body pain diagram	x	x	x	x	x	
PROMIS pain interference short form	x	x	x	x	x	
KIDSCREEN-27	x					x
PROMIS sleep interference	x					x
PROMIS sleep disruption	x					x
Checklist of care for CP in the preceding month	x					x
Health chart review	x					x

### Statistical analyses

We performed descriptive analyses and feasibility indicators were measured. For process indicators, we calculated frequency counts for participants who: contacted HS for information; attended the in-person screening meeting; and met eligibility requirements. The follow-up rate for each time-point was measured. We also reported voluntarily provided reasons for non-participation or study withdrawal. Resource indicators were assessed by measuring data completion at each time-point.

The management indicator was assessed by summarizing baseline characteristics using means and standard deviations for normally distributed continuous variables, medians and interquartile ranges for non-normally distributed continuous variables, and proportions for categorical variables. Spaghetti plots were used to visualize short-term pain trajectories to estimate variability as an indicator for the planned larger cohort study. The KIDSCREEN-27 and PROMIS raw scores were rescaled to standardized T-scores with a mean of 50 and standard deviation of 10. Scores > 50 indicate a negative impact for PROMIS measures and a better score on KIDSCREEN-27 domains. The data analysis was generated using SAS software, Version 9.4, Copyright © 2016 SAS Institute Inc. SAS and all other SAS Institute Inc. product or service names are registered trademarks or trademarks of SAS Institute Inc., Cary, NC, USA.

## Results

### Feasibility indicators

#### Study processes

**Fig. 2:**
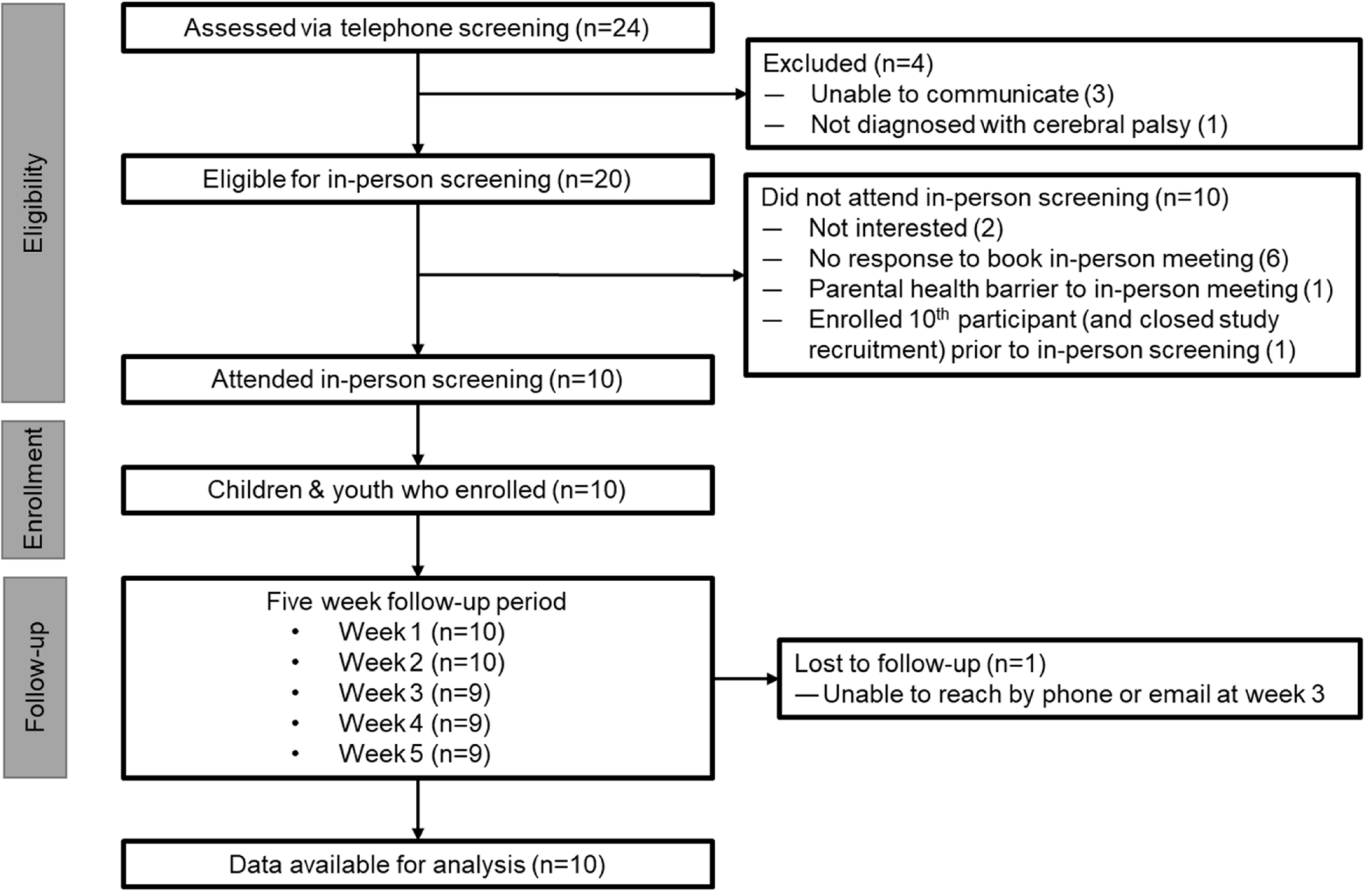
STROBE flow chart (STROBE: Strengthening the Reporting of Observational Studies in Epidemiology). Data completeness: Baseline=100%, week 1= missing one item response; week 2=100%; Week 3= only FPS-R reported by one participant and no data for another; Week 4 & 5: loss to follow-up of one participant

The lead author contacted 24 eligible children and their parents/guardians (Fig. 2). All Holland Bloorview participants were recruited using the research call list. Grandview participants were recruited through social media (n=1) or referred by clinicians (n=4). The recruitment rate for those contacted by phone, who met the initial eligibility criteria and were screened in-person was 50% (10/20). All who attended in-person screening were enrolled (10/10). The follow-up rate was 100% for weeks one and two, and 90% for weeks three to five.

Feedback: Some (n=3) questioned their eligibility because they weren’t experiencing pain and wording on the study flyers wasn’t clear. One individual stated the eligibility criteria excluded those unable to verbally communicate or complete electronic questionnaires independently (.e.g. those who could vocalize and needed someone to manually record responses).

#### Resources indicator

Nine participants chose the electronic questionnaire over paper. Participants completed the baseline questionnaire in 20-30 minutes. The first to fourth follow-ups took less than 10 minutes and the fifth follow-up took less than 15 minutes to complete. Close monitoring and telephone reminders to complete follow-ups was required for 60% of participants. Data completion was: 100% for all baseline variables; 100% for follow-up week 1 except for one missing FPS-R response; 100% for follow-up week 2; missing from one participant for follow-up week 3 (except the FPS-R) and another did not respond to follow-ups three through five. Other important measures for the future cohort study, including sleep characteristics, physical and psychological well-being, had no missing values at baseline and were missing only for one participant at follow-up five.

Unavailable data was common in health records: 40% for hip imaging, 40% for gastro-intestinal disorders, 50% for epilepsy/seizure disorders, 50% for mental health disorders, and 50% for no medication listed within the past year.

Participant feedback: No problems were reported regarding the emailed questionnaire links and electronic questionnaires were easy to complete.

#### Management indicator

The age of participants ranged from 8-17 years, the female to male ratio was 1, and GMFCS levels ranged from I-IV (Table 3). Mean baseline pain intensity was 2.4/10 (SD=1.4) on the FPS-R and 80% reported pain in the preceding week. Eighty percent of participants reported doing home exercises for CP-related care in the month preceding baseline, 80% wore a brace, 40% had physiotherapy, 40% had massage therapy, and 10% received botulinum toxin injections.

**Table 3:** Baseline characteristics of study participants (n=10)

Characteristic	
Age in years, mean (SD); median (IQR)	11.6 (3.5); 12.0 (7)
CP type, n (%)	
Spastic	8 (80%)
Other (Ataxic, Dystonic, Mixed)	2 (20%)*
Limb involvement, n (%)	
Hemiplegic, Triplegic, Quadriplegic	5 (50%)*
Diplegic	5 (50%)
GMFCS level, n (%)	
I, II, IV	5 (50%)*
III	5 (50%)
V	0
Pain intensity, mean (SD); median (IQR)	
FPS-R	2.8 (2.9); 3.0 (4)
NRS	2.3 (2.5); 1.5 (4)
Pain interference^#^, mean (SD)	46.6 (8.6)
KIDSCREEN-27, mean (SD)	
Physical well-being	46.8 (8.2)
Psychological well-being	52.2 (10.8)

*Categories with less than five participants were aggregated to avoid inadvertent identification

FPS-R: Faces Pain Scale-Revised; GMFCS: Gross Motor Function Classification System; IQR: interquartile range; NRS: Numeric Rating Scale; SD: standard deviation

^#^PROMIS Pediatric Short Form v2.0 – Pain Interference 8a

**Fig. 3:**
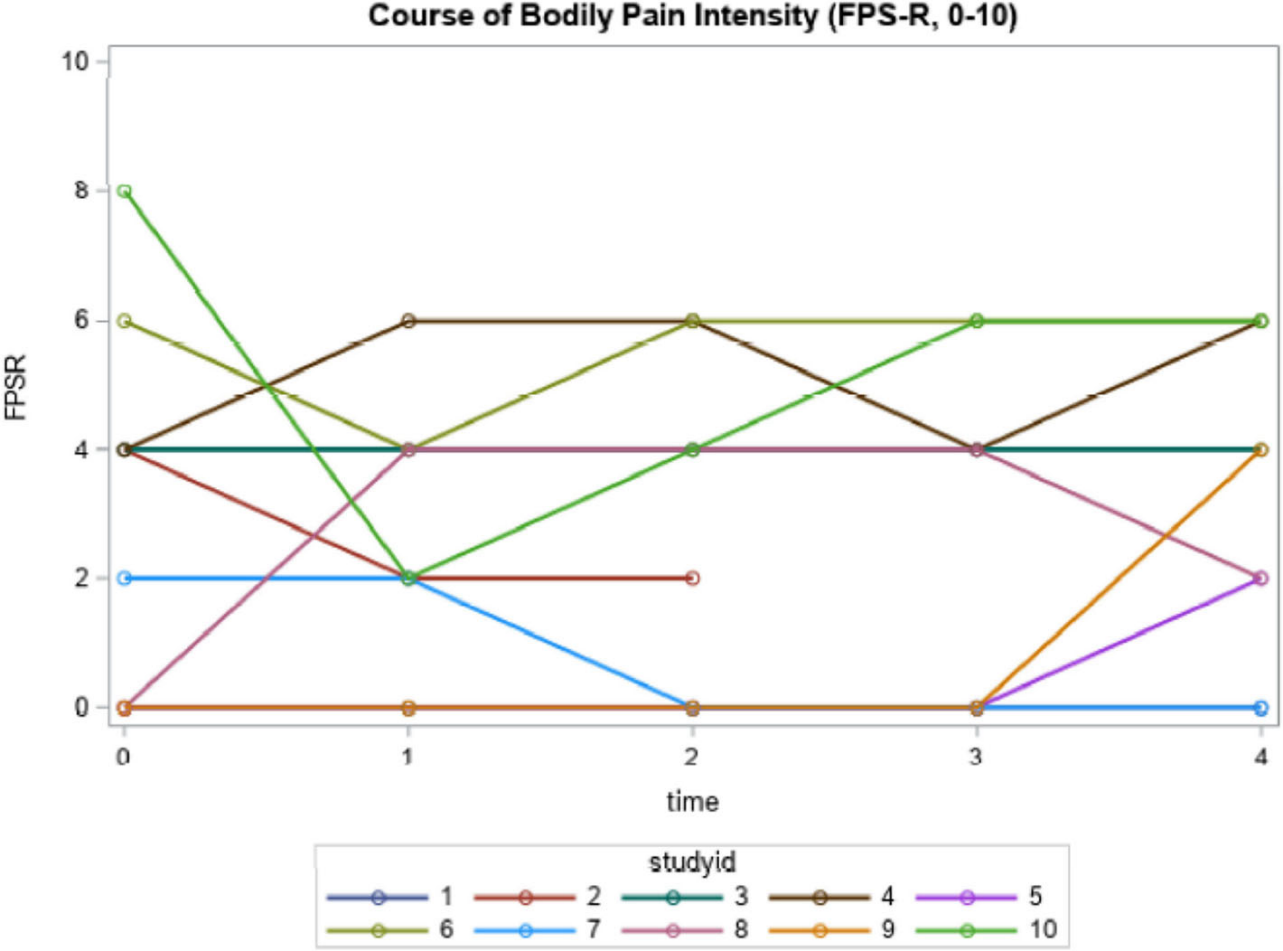
Individual course of self-reported pain intensity using the FPS-R (n=10)

**Fig. 4:**
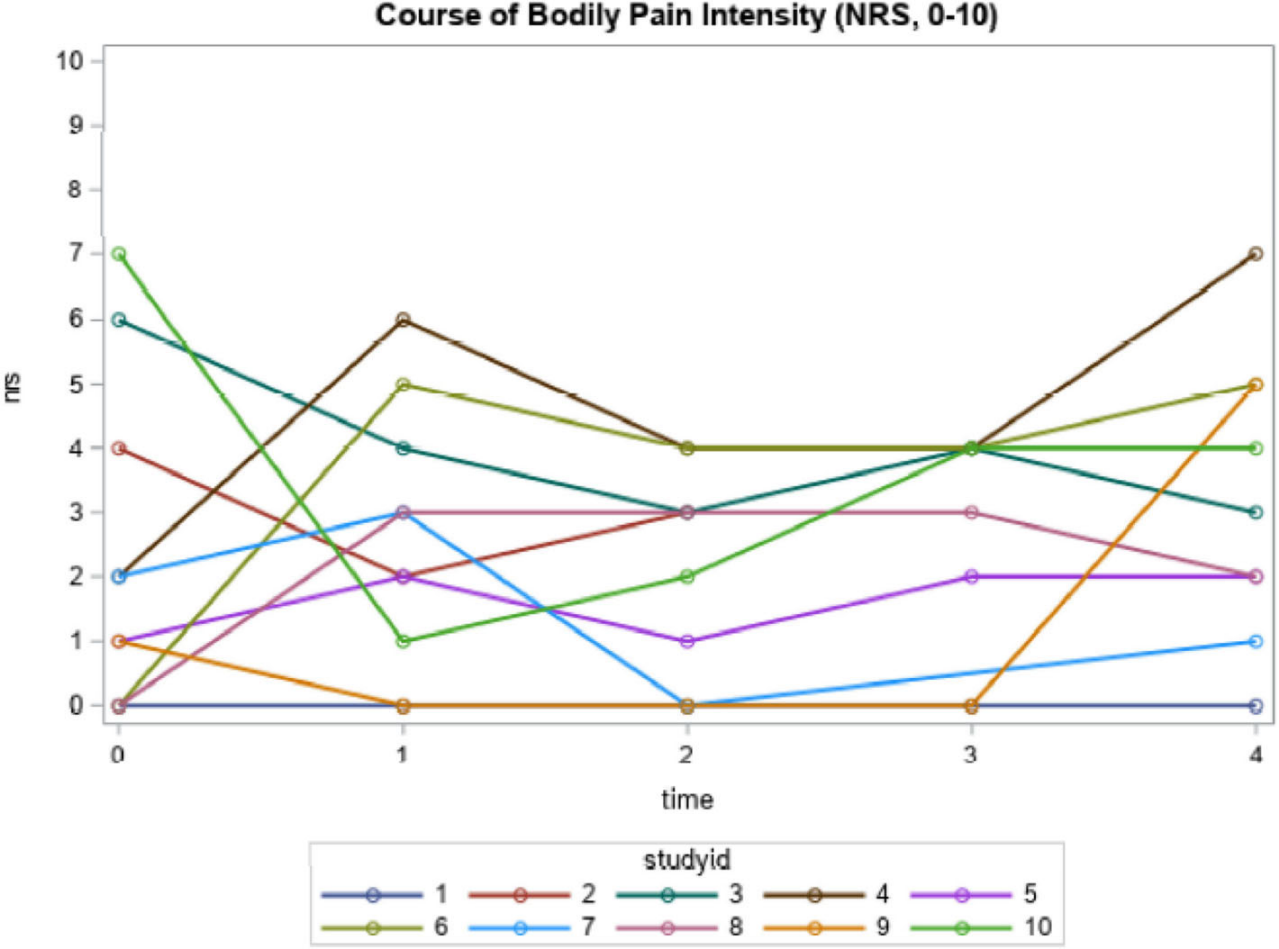
Individual course of self-reported pain intensity using the NRS (n=10)

Participants reported different pain trajectories suggesting that pain did not change for some while it was fluctuating, increasing or decreasing for others during follow-up (Figs. 3 and 4). Mean pain intensity for all participants ranged from *x̅*=2.6 (2.5) to *x̅*=3.3 (2.5) on the FPS-R (baseline to follow-up 4), however individual FPS-R scores ranged from 0-8/10 at baseline and 0-6/10 over the follow-ups. The mean NRS scores ranged from *x̅*=2.0 (1.6) to *x̅*=3.2 (2.2). Individual scores ranged from 0/10 to 7/10 across the time-points.

Mean pain interference (PROMIS PI) T-score across time-points ranged from *x̅*=44.0 (9.4) to *x̅*= 48.5 (9.4). Individual T-scores were as low as 34 across all time-points for one participant and up to 56.8 (with a range of 51.7 to 62.4) for another individual.

No adverse events related to mood were reported and no participants had KIDSCREEN-27 psychological well-being domain raw scores below 21/35.

## Discussion

This pilot study demonstrated that this methodology can be successfully implemented in a larger cohort study and that it will be feasible to measure short-term pain trajectories and their association with physical and psychological well-being in children and youth with CP. The results suggest that our recruitment and data collection methodologies were successful. Nevertheless, components of our methodology can be improved.

The process feasibility indicator identified some limitations with our recruitment methodology. Our recruitment rate (50%) suggests the potential for selection bias in the future cohort study. We received feedback regarding uncertainty related to study eligibility by individuals without pain, indicating that our eligibility criteria should be stated more clearly. Moreover, we did not recruit participants with a GMFCS level V. In our future cohort study, eligibility criteria should include those who use alternate communication methods such as eye gaze or vocalizations and who may require assistance to physically complete questionnaires. Additionally, we will consider stratified recruitment approaches to ensure better representation across all five GMFCS levels. These changes may enable participation of those with greater motor impairment. There is evidence of a positive association between pain and increasing age among children with CP [4, 5, 28]. Thus, a stratified recruitment method based on age may also be appropriate. Despite these limitations, recruitment was accomplished quickly, with good follow-up rates and only one loss-to-follow-up. We also engaged stakeholders (youth) with CP to provide guidance regarding study processes. We will repeat this in our future cohort study by creating a stakeholder advisory committee including youth with CP, parents/guardians of individuals with CP and clinicians. Finally, we used self-report measures that were easy to complete online.

One resources indicator clarified the manpower needed to ensure adequate data completion. Most participants required both automated and telephone reminders to complete questionnaires. Also, the proportion of non-reported or missing data from chart reviews for hip formation/migration, gastro-intestinal disorders such as constipation, use of feeding tubes or gastro-esophageal reflux suggest that capturing these confounders may be problematic in our future cohort study. These are potential sources of pain that may impact daily function and well-being deleteriously. Adding self-reported items and conducting sensitivity analyses comparing model results for those with complete confounder data to those with missing/not reported data would be helpful.

The management indicator suggested that our study was successful in capturing variability in pain intensity. Pain was commonly experienced by participants, concurring with previous studies [1, 6, 29]. Pain intensity varied over time and between the children, providing support for conducting a larger cohort study to assess pain trajectories. We will investigate in a larger sample how pain intensity varies in this group. Based on published evidence, clinical and lived experience, we hypothesize there may be as many as five distinct pain trajectories (stable, fluctuating, increasing, decreasing, and no pain). Some individuals do not report experiencing pain, while others’ pain intensity levels may be influenced (increased, decreased, fluctuating or stable) by comorbidities or ongoing interventions (e.g. medications, surgery, Botox injections, bracing, etc.) [4, 6, 7]. The pilot study results also indicate that pain interference varied within and between participants. Finally, there were no adverse events related to self-reported low mood during the course of this study.

### Future research

Our future cohort study will build on the knowledge gained in this pilot study and will aim to identify short-term pain trajectories as prognostic indicators of short-term physical and psychological well-being. We will explore relationships between pain intensity, pain interference, well-being, and sleep as a means of providing a more holistic approach to understanding the support needs of these children and youth. In our future study, we will use Monte Carlo simulation methods as described by Dziak et al. (2014) to estimate our required sample size [30].

## Conclusions

This pilot study provides evidence and insight to support a larger, multi-site cohort study to identify pain trajectories and their association with physical and psychological well-being in children and youth with cerebral palsy.

## Data Availability

The datasets generated and/or analysed during the current study are not publicly available due to the small sample size and our duty to avoid inadvertent identification of individuals, but are available from the corresponding author on reasonable request.

## Abbreviations

CP: Cerebral palsy
CTC: Children’s treatment center
GMFCS: Gross Motor Function Classification System
FPS-R: Faces Pain Scale-Revised
NRS: Numeric Rating Scale
STROBE: Strength of Reporting Observational Studies in Epidemiology

## References

[1] Oskoui M, Coutinho F, Dykeman J (2013). An update on the prevalence of cerebral palsy: a systematic review and meta-analysis. Dev Med Child Neurol.

[2] Rosenbaum P, Paneth N, Leviton A (2007). A report: the definition and classification of cerebral palsy April 2006. Dev Med Child Neurol Suppl.

[3] Dewan T, Cohen E (2013). Children with medical complexity in Canada. Paediatr Child Health.

[4] Eriksson E, Hägglund G, Alriksson-Schmidt AI (2020). Pain in children and adolescents with cerebral palsy - a cross-sectional register study of 3545 individuals. BMC Neurol.

[5] McKinnon CT, Meehan EM, Harvey AR (2019). Prevalence and characteristics of pain in children and young adults with cerebral palsy: a systematic review. Dev Med Child Neurol.

[6] Penner M, Xie WY, Binepal N (2013). Characteristics of pain in children and youth with cerebral palsy. Pediatrics..

[7] Christensen R, MacIntosh A, Switzer L (2017). Change in pain status in children with cerebral palsy. Dev Med Child Neurol.

[8] Colver A, Rapp M, Eisemann N (2015). Self-reported quality of life of adolescents with cerebral palsy: a cross-sectional and longitudinal analysis. Lancet..

[9] Dickinson HO, Parkinson KN, Ravens-Sieberer U (2007). Self-reported quality of life of 8-12-year-old children with cerebral palsy: a cross-sectional European study. Lancet..

[10] Westbom L, Rimstedt A, Nordmark E (2017). Assessments of pain in children and adolescents with cerebral palsy: a retrospective population-based registry study. Dev Med Child Neurol.

[11] van der Slot WM, Nieuwenhuijsen C, Van Den Berg-Emons RJ (2012). Chronic pain, fatigue, and depressive symptoms in adults with spastic bilateral cerebral palsy. Dev Med Child Neurol.

[12] Parkes J, White-Koning M, Dickinson HO (2008). Psychological problems in children with cerebral palsy: a cross-sectional European study. J Child Psychol Psychiatry.

[13] Birnie KA, Hundert AS, Lalloo C (2019). Recommendations for selection of self-report pain intensity measures in children and adolescents: a systematic review and quality assessment of measurement properties. Pain..

[14] Thabane L, Ma J, Chu R (2010). A tutorial on pilot studies: the what, why and how. BMC Med Res Methodol.

[15] Harris PA, Taylor R, Minor BL (2019). The REDCap consortium: Building an international community of software platform partners. J Biomed Inform.

[16] Harris PA, Taylor R, Thielke R (2009). Research electronic data capture (REDCap)--a metadata-driven methodology and workflow process for providing translational research informatics support. J Biomed Inform.

[17] Hicks CL, von Baeyer CL, Spafford PA (2001). The Faces Pain Scale-Revised: toward a common metric in pediatric pain measurement. Pain..

[18] Miro J, Huguet A (2004). Evaluation of reliability, validity, and preference for a pediatric pain intensity scale: the Catalan version of the faces pain scale--revised. Pain..

[19] Bieri D, Reeve RA, Champion GD (1990). The Faces Pain Scale for the self-assessment of the severity of pain experienced by children: development, initial validation, and preliminary investigation for ratio scale properties. Pain..

[20] Castarlenas E, Jensen MP, von Baeyer CL (2017). Psychometric Properties of the Numerical Rating Scale to Assess Self-Reported Pain Intensity in Children and Adolescents: A Systematic Review. Clin J Pain.

[21] Dworkin RH, Turk DC, Farrar JT (2005). Core outcome measures for chronic pain clinical trials: IMMPACT recommendations. Pain..

[22] Cunningham NR, Kashikar-Zuck S, Mara C (2017). Development and validation of the self-reported PROMIS pediatric pain behavior item bank and short form scale. Pain..

[23] Varni JW, Stucky BD, Thissen D (2010). PROMIS Pediatric Pain Interference Scale: an item response theory analysis of the pediatric pain item bank. J Pain.

[24] Northwestern University. PROMIS Pediatric Short Form v2.0 - Pain Interference 8a 2018 [Available from: http://www.healthmeasures.net/index.php?option=com_instruments&view=measure&id=711&Itemid=992.

[25] Ravens-Sieberer U, Herdman M, Devine J (2014). The European KIDSCREEN approach to measure quality of life and well-being in children: development, current application, and future advances. Qual Life Res.

[26] Natalucci G, Bucher HU, Von Rhein M (2017). Population based report on health related quality of life in adolescents born very preterm. Early Hum Dev.

[27] Forrest CB, Meltzer LJ, Marcus CL, et al. Development and validation of the PROMIS Pediatric Sleep Disturbance and Sleep-Related Impairment item banks. SleepJ. 2018;41(6):1-13. 10.1093/sleep/zsy054 29546286

[28] Parkinson KN, Dickinson HO, Arnaud C (2013). Pain in young people aged 13 to 17 years with cerebral palsy: cross-sectional, multicentre European study. Arch Dis Child.

[29] Ostojic K, Paget S, Kyriagis M (2020). Acute and Chronic Pain in Children and Adolescents With Cerebral Palsy: Prevalence, Interference, and Management. Arch Phys Med Rehabil.

[30] Dziak JJ, Lanza ST, Tan X (2014). Effect Size, Statistical Power and Sample Size Requirements for the Bootstrap Likelihood Ratio Test in Latent Class Analysis. Struct Equ Model.

